# Markerless gene deletion in *Ralstonia solanacearum* based on its natural transformation competence

**DOI:** 10.3389/fmicb.2022.977580

**Published:** 2022-09-13

**Authors:** Jinli Yan, Nuoqiao Lin, Xiaoqing Wang, Xuemei Chen, Huishan Wang, Qiqi Lin, Xiaofan Zhou, Lianhui Zhang, Lisheng Liao

**Affiliations:** ^1^Guangdong Province Key Laboratory of Microbial Signals and Disease Control, Integrative Microbiology Research Centre, South China Agricultural University, Guangzhou, China; ^2^Guangdong Laboratory for Lingnan Modern Agriculture, South China Agricultural University, Guangzhou, China

**Keywords:** *Ralstonia solanacearum*, fusion PCR, natural transformation, FLP/*FRT*, gene deletion

## Abstract

*Ralstonia solanacearum* species complex (RSSC) is a group of Gram-negative bacterial pathogen capable of infecting numerous plants and crops, causing severe vascular wilt diseases. Functional analysis of the genes associated with bacterial virulence is critical for elucidating the molecular mechanisms that govern the bacterial pathogenicity. To this end, an efficient gene deletion method would be of great help. In this study, we set to develop an efficient and simple markerless gene deletion method by exploiting its natural transformation competence and the FLP/*FRT* recombination system. We found that natural transformation using PCR products provided much higher transformation frequency than the plasmid-based triparental mating and electroporation. We thus generated the gene deletion fusion PCR fragments by incorporating the upstream and downstream DNA fragments of the target gene and an antibiotic resistance gene flanked by *FRT* sites, and delivered the PCR products into *R. solanacearum* cells through natural transformation. Using this method, we knocked out the *epsB* and *phcA* genes, which are associated with exopolysaccharide (EPS) biosynthesis and regulation, respectively, in several *R. solanacearum* strains isolated from different host plants at a frequency from 5 (1E-08) to 45 (1E-08). To remove the antibiotic marker gene, the plasmid expressing the FLP enzyme was introduced into the above knockout mutants, which enabled removal of the marker gene. The effective combination of natural transformation and the FLP/*FRT* recombination system thus offers a simple and efficient method for functional study of putative virulence genes and for elucidation of *R. solanacearum* pathogenic mechanisms.

## Introduction

Bacterial wilt caused by *Ralstonia solanacearum* species complex (RSSC) is known as one of the most important plant bacterial diseases (Genin and Denny, 2012). These soil-borne pathogenic bacteria cause vascular wilt on more than 400 plant species over 50 families including numerous *solanaceous* crops such as tomato, potato, tobacco and eggplant, resulting in severe economic losses (Flavier et al., 1997; Saile et al., 1997; Alvarrez et al., 2019). The pathogen can survive in soil or water environment for many years, and is widely distributed in tropical, subtropical, and warm temperate regions of the world (Hayward, 1991). When encountering susceptible host plants, *R. solanacearum* invades root xylem vessels, colonizes and moves rapidly in the plant through vascular tissues (Monteiro et al., 2012). The pathogen produces an array of virulence factors, including extracellular polysaccharide (EPS), cell wall-degrading enzymes (CWDE), and type III secretion system, which collectively contribute to its virulence (Hayward, 1991). Among them, extracellular polysaccharide EPS I is known as a major virulence factor of *R. solanacearum* (Orgambide et al., 1991). In the process of rapid colonization, the pathogen colonizes in the aerial parts of the plant and produces massive amounts of exopolysaccharides (EPSs), which obstruct water transport resulting in wilt symptoms and death of plants (Saile et al., 1997).

The heterogeneity of RSSC strains is mainly due to their capacity for natural transformation and recombination (Coupat-Goutaland et al., 2011; Wicker et al., 2012). *Ralstonia solanacearum* is among the about 40 bacterial species that are known to be naturally transformable by taking up free foreign DNA (Lorenz and Wackernagel, 1994). It was shown that *R. solanacearum* could take up large DNA fragments ranging from 30 to 90 kb by DNA replacement (Coupat et al., 2008), and a minimum of 50 bp of linear homologous DNA is sufficient for integrating into the bacterial genome (Bertolla et al., 1997). The competence for natural transformation may provide microorganisms an evolution mechanism for adaptation to various environmental conditions, and this property could also be exploited for genetic manipulation and functional analysis.

Genetic knockout or gene deletion techniques play an essential role in characterization of the genes associated with pathogenesis and for elucidating the regulatory networks governing the microbial physiology and virulence. In *R. solanacearum,* common methods for gene deletion are based on homologous recombination exchange using triparental or biparental mating or electroporation to introduce constructed plasmid containing homologous fusion fragment with a marker gene for facilitating selection (Genin and Denny, 2012). In this study, we present here a simple and efficient method for stable and directed deletion of genes in *R. solanacearum* by exploiting its natural transformation competence. In this method, the fusion DNA fragments homologous to the upstream and downstream DNA sequences of the target gene containing an antibiotic resistance marker gene in the middle was generated by fusion PCR and introduced into the bacterial cells through natural transformation, thus avoiding the tedious steps in construction and purification of plasmid. In addition, the marker gene can be easily eliminated as *FRT* sequences could be incorporated to the right and left borders of the maker gene, respectively, which can be recognized and cut off by the FLP enzyme (Volkert and Broach, 1986; Zhu and Sadowski, 1995; Ishikawa and Hori, 2013). To our knowledge, this is the first report on developing such a PCR fragment-based marker-free gene deletion method in *R. solanacearum.* To validate this method and for the convenience of phenotype observation, we selected two genes associated with the bacterial virulence as the target genes, including *phcA* that encodes a global regulator involved in regulation of virulence factor biosynthesis such as EPS and type III secretion system (Huang et al., 1995, 1998), and *epsB* that is essential for the production of the key virulence factor EPS I (Hayashi et al., 2019). We found that this method is simple and efficient, which should be useful for gene deletion or integration of a foreign DNA fragment into the bacterial chromosome.

## Materials and methods

### Bacterial strains and culture conditions

The bacterial strains and plasmids used in this study are listed in Supplementary Table S1. *Escherichia coli* strain was grown at 37°C in Luria Bertani (LB) broth medium (10 g tryptone, 5 g yeast extract, and 10 g NaCl, pH 7.0). *R. solanacearum* strains were cultured at 28°C for overnight in CTG rich broth (10 g tryptone, 5 g glucose, and 1 g casamino acids) or minimal medium (MM; K_2_HPO_4_ 10.5 g/L, KH_2_PO_4_ 4.5 g/L, (NH_4_)_2_SO_4_ 2.0 g/L, MgSO_4_·7H_2_O 0.2 g/L, CaCl_2_ 0.01 g/L, FeSO_4_ 0.005 g/L, MnCl_2_ 0.002 g/L, mannitol 2.0 g/L, glycerol 2 g/L). The cellulase activity assay agar contains 1.0 g carboxymethyl ethyl cellulose, 3.8 g sodium phosphate, 8.0 g agarose per liter. Antibiotics were added at the following concentrations: kanamycin (Km) 50 mg/L; gentamicin (Gm), 50 mg/L; and rifampicin (Rif), 25 mg/L. The Rif-resistant spontaneous mutants of *R. solanacearum* strains were obtained by streaking corresponding bacterial cells on the CTG plate supplemented with Rif at a final concentration of 25 μg/L.

### PCR amplification of DNA fusion fragments for gene deletion

**Figure 1:**
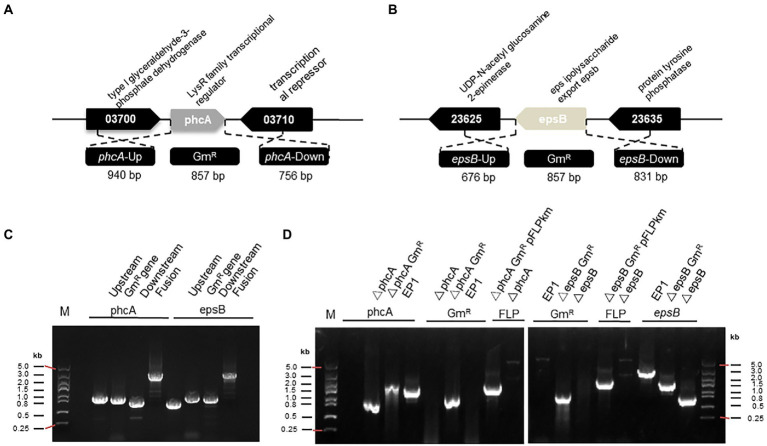
PCR fragment-based gene mutation in *Ralstonia solanacearum* strain EP1. **(A)** The genetic organization *of phcA* and flanking genes. **(B)** The genetic organization *of epsB* and its flanking genes. **(C)** Generation of fusion DNA fragments by PCR for replacement of *phcA* and *epsB*, respectively. **(D)** PCR analysis for validation of *phcA* and *epsB* deletions in *R. solanacearum* EP1. Symbol: *phcA*-Up, the left flanking region of *phcA*; Gm^R^, gentamicin resistance gene; *phcA*-Down, the right flanking region of *phcA*; *epsB*-Up, the left flanking region of *epsB*; *epsB*-Down, the right flanking region of *epsB*; Fusion, PCR fragment containing the flanking regions of the target gene and Gm^R^; pFLPkan, a plasmid containing FLP recombinase gene; EP1, *R. solanacearum* wild type.

The primers used in this study were listed in Supplementary Table S2. As illustrated in Figure 1A, a 940 bp upstream DNA fragment (*phcA*-Up) and a 756 bp downstream DNA fragment (*phcA*-Down) of the *phcA* gene, were amplified by PCR using the primer pairs phcA-1/phcA-2 and phcA-5/phcA-6, respectfully, with *R. solanacearum* EP1 genomic DNA as the template. To facilitate screening of transformants and subsequent removal of the mark gene, the 925 bp of the *gen* coding sequence containing the *FRT* sequence (GAAGTTCCTATTCTCTAG-AAAGTATAGGAACTTC) at the 5′ and 3′ ends, which is FLP recombinase recognition site (Yan et al., 2016), was amplified by PCR using the primer pair phcA-3/phcA-4 with pBBRI-MCS5 plasmid DNA as a template. The primer phcA-3 contains 10 bp overlapping nucleotides at 5′-end homologous to the *phcA-2* for fusion with the phcA-Up and 18 bp nucleotides at 3′-end for amplification of the Gm resistance gene (*gen*), and the primer phcA-4 contains 19 bp nucleotides at 3′-end for amplification of *gen* and 12 bp nucleotides at 5′-end homologous to the *phcA-5* for fusion with the phcA-Down. And then, the *phcA* fusion fragment was amplified using *phcA*-Up, Gen, Down as templates using the primer pair phcA-1/phcA-6. The PCR reactions were conducted with the DNA polymerase (2 × T5 super PCR mix, Qing Ke) using standard conditions (98°C pre degeneration 5 min, 98°C denaturing 30 s, 60°C or 55°C annealing 30 s, 72°C elongation 1 min, an elongation time of approximately 1 min per kb of PCR product and 72°C elongation again 5 min). The PCR products were recovered or purified using E.Z.N.A.® Gel Extraction Kit/Cycle Pure Kit (Omega Company). The fusion fragment of the *epsB* gene (Figure 1B) was amplified in a similar way using the primer pairs described in Supplementary Table S2.

### Transformation by triparental mating

Triparental mating was conducted following the method describing previously. In brief, the suicide plasmid pK18mobsacB (Schäfer et al., 1994) was transformed into *E. coli* DH5α competent cells following the manufacturer’s protocol (Life Technologies Corporation, Beijing, China). The bacterial cells were cultured at 37°C with shaking for 1 h, and the transformants were selected in LB medium supplemented with Gm and verified by PCR analysis. The plasmid was mobilized into *R. solanacearum* by using the pRK2013-based triparental mating as described previously (Ditta et al., 1980) with minor modifications. In brief, the donor, helper and recipient strains were inoculated in LB and CTG plates containing Gm or Km and Rif, respectively, at 37°C or 28°C overnight, which were resuspended with corresponding liquid media and mixed evenly at 1:0.5:1 ratio before spreading gently on the CTG plate. After incubation 24 h, transformants were then selected on the CTG plate containing antibiotics Gm and Rif at 28°C for 3 days, and verified by PCR amplification using the primers pK18-F/ pK18-R listed in Supplementary Table S2.

The number of recipient cells used for mating was determined by counting colony forming units (CFU) on CTG plate with series dilutions. Plates were incubated at 28°C, and then the numbers of recipient cells were recorded after 3 days. Transformation frequency was calculated as the number of transformants observed per recipient cell applied.

### Transformation by electroporation

Preparation and electroporation of *R. solanacearum* competent cells were conducted as previously described (Zhang et al., 2014) with minor modifications. For preparation of the electrocompetent cells, *R. solanacearum* cells were cultured in CTG liquid medium at 28°C for 24 h and cooled in ice bath for about 15 min. The bacterial cells were harvested by centrifuging 1 ml of culture at 5000 rpm for 5 min at 4°C. The bacterial pellets were washed and resuspended twice consecutively with 1 ml of ultrapure water and 1 ml of water containing 10% glycerol. The bacterial pellets were then resuspended in 100 μl of 10% glycerol to obtain the electrocompetent cells.

For electroporation, about 1 μg of plasmids were added to 100 μl electrocompetent cells and mixed gently before being transferred to an electroporation cup (1 mm, BioRad). Electroporation was conducted at 2.5 kV for 5 ms, and then 700 μl CTG liquid medium were added into the electroporated cells, which were allowed to revive at 28°C for 24 h. The transformants were selected on CTG plates supplemented with Gm and Rif, and verified by PCR amplification using the primers pK18-F/pK18-R listed in Supplementary Table S2.

The number of electrocompetent cells used faor electroporation was determined in the same way as triparental mating. Transformation frequency was calculated as the number of transformants observed per electrocompetent cell applied.

### Natural transformation

Natural transformation of *R. solanacearum* was performed similar to that described previously (Monteiro et al., 2012). *Ralstonia solanacearum* cells were grown at 28°C for 2 days in MM supplemented with 10% glycerol. An aliquot of 100 μl cell cultures were taken and mixed gently with about 1 μg of plasmid pK18mobsacB containing a Gm resistance gene, which were spread on a 25-mm, 0.45-μm pore size cellulose nitrate membrane laid on the surface of a CTG medium agar plate and incubated at 28°C for 24 h. The bacterial cells were then resuspended with 500 μl of sterile distilled water and 100 μl cells were spread on a CTG-agar plate supplemented with Gm to select transformants. The method of transformation frequency was based on Coupat’s experimental method (Coupat et al., 2008), which was calculated as the number of transformants observed per *R. solanacearum* cell applied.

For generation of knockout mutants, DNA fusion fragments for homologous knockout of *phcA* and *epsB* were prepared by PCR amplification as described above. The PCR fragments were introduced into *R. solanacearum* cells using the natural transformation procedures described above. Deletion mutants containing Gm resistance marker were validated by PCR amplifications using the primer pairs phcA-check-F/phcA-check-R and epsB-check-F/epsB-check-R, respectively.

### Elimination of the Gm resistance gene in *Ralstonia solanacearum* mutants

In order to obtain deletion mutants without selective marker, we choose the FLP/*FRT* system to remove the Gm resistance gene (Abremski et al., 1983; Yan et al., 2016). When we synthesized primers, the *FRT* sequences were added to the primers phcA-3/phcA-4 and epsB-3/epsB-4. The FLP gene was commercially synthesized (QingKe), and cloned into the plasmid pBBR1-MCS2 under the control of the P*gdh* promoter to generate the expression construct pFLP_Km_ (Supplementary Figure S4). FLP is a site-specific recombinase, which can specifically recognize the *FRT* sites and remove the DNA sequences (the Gm resistance gene in this case) between the two FRT sites. The plasmid pFLP_Km_ was then transferred to *E. coli* by thermal shock at 42°C and stored at −80°C. The plasmid pFLP_Km_ was introduced into the competent cells of *R. solanacearum* knockout mutants by using electroporation. Transformants were preliminarily selected through the Km resistance carried by the construct pFLP_Km_, and verified by PCR using the primers MCS-F/MCS-R. Km resistance colonies were then sub-streaked on the CTG solid plates supplemented with Gm or Km, respectively. Colonies without the Gm resistance but maintaining Km resistance were deemed as the mark-free deletion mutants and validated by PCR amplifications using the primers phcA-check-F/phcA-check-R and epsB-check-F/epsR-check-R (Supplementary Figure S6), respectively. Finally, Km resistance mark-free deletion mutants were subcultured 1–4 times in liquid CTG medium without antibiotics for discarding plasmid pFLP_km_. The subculture were then diluted and spread on the CTG solid plates supplemented with Rif, and the producing colonies verified by PCR using the primers MCS-F/MCS-R for confirming loss of the plasmid.

### Cellulase activity assay

Assay of cellulase activity was conducted following the method described previously (3) with minor modifications (Chatterjee et al., 1995). Briefly, *R. solanacearum* strains were inoculated in 10 ml CTG liquid medium and cultured till optical density at 600 nm (OD_600_) reached about 1.5. 20 μl to be tested cultures were added into wells in the cellulase activity assay plate. The plates were incubated at 28°C for 24 h, stained with 20 ml 0.1% Congo red(wt/vol)for 15 min, and then socked with about 40 ml 1 mol/L NaCl for 10 min, and repeated soak one more time. The NaCl solution was poured off, and the diameter of clear zone was observed and measured. The experiment was repeated three times.

### EPS production assay

Quantification of EPS production was performed as previously reported with minor modifications (Chen and Huang, 2019; Shen et al., 2020). In brief, *R. solanacearum* was cultured in CTG solid medium at 28°C for 2 day, which were washed and resuspended with 2 ml of sterile distilled water. And then the suspension liquid was centrifuged at 12,000 rpm for 10 min. The supernatants were collected and mixed with 2 volumes of absolute ethanol, and the mixtures were incubated at 4°C overnight. The precipitated EPS was isolated by centrifugation and dissolved in 200 μl sterile water. An aliquot of 50 μl EPS sample was added to a well of the 96-well plate, to which 150 μl concentrated sulfuric acid and 30 μl phenol (5%, wt/vol) were added consecutively for color development. The relative sugar content in the EPS sample was determined by measuring OD_490_.

### Pathogenicity assay

The eggplant seedlings were potted till fully expanded 3–4 leaves. Before inoculation, the eggplant seedlings were pulled out from soil to generate wounds in roots and then replanted in soil. About 10 ml of fresh *R. solanacearum* cultures in CTG broth (OD 600 ≈ 1.5) were added evenly into the soil close to wounded eggplants. The inoculated plants were then grown in an 28°C incubator with 14 h light and 10 h dark cycle. Each treatment contained 10 plants and the experiment was repeated three times. The disease symptoms were observed and recorded daily on a disease index scale from 0 (no wilt), 1 (1%–25% leave wilted), 2 (26% ~ 50% leave wilted), 3 (51% ~ 75% leave wilted), 4 (>75% leave wilted), and 5 (plant died; Yang et al., 2017).

## Results

### Efficiency of *Ralstonia solanacearum* natural transformation

**Figure 2:**
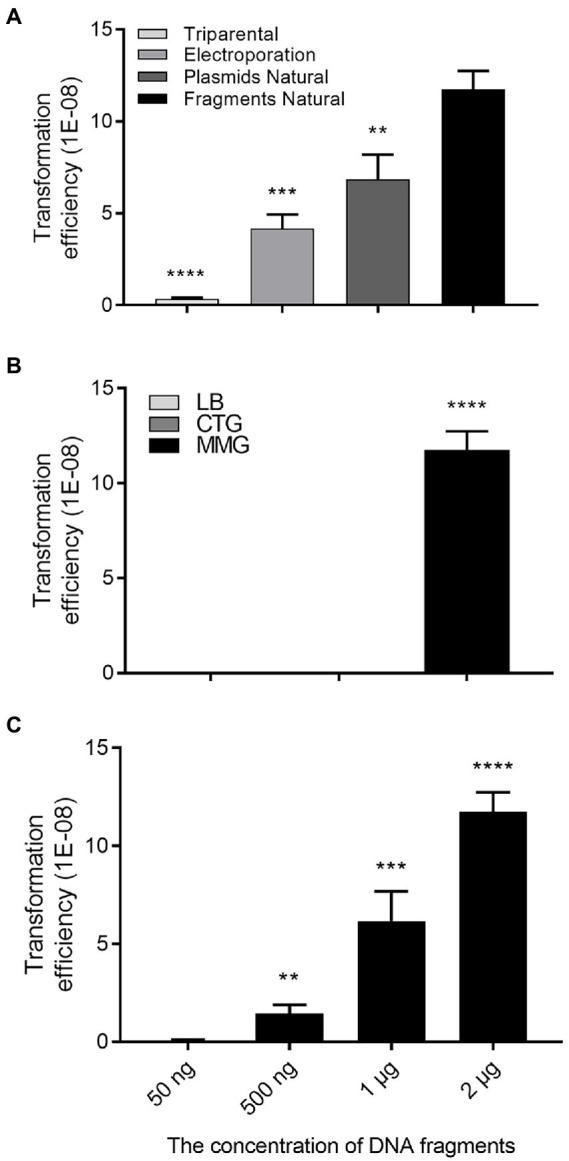
Transformation efficiency assay. **(A)** Transformation efficiency of three approaches, i.e., triparental mating, electroporation, and natural transformation plasmid and linearized plasmid, respectively. The same DNA concentration (~1 μg) were used for electroporation or natural transformation. **(B)** Natural transformation efficiencies of the bacterial cells cultured in LB, CTG, and MMG media, respectively. **(C)** Influence of DNA concentration on natural transformation efficiency. Transformation frequency was calculated as the number of transformants observed per *Ralstonia solanacearum* cell applied. Data shown are means ± standard deviations (SD) of three independent experiments with triplicates. Statistical significance: ^**^p-value < 0.01; ^***^p-value < 0.001; ^****^p-value < 0.0001 (unpaired *t*-test).

To delete or knock out target genes in *R. solanacearum*, we used to employ triparental matting or electroporation to deliver the plasmid containing homologous DNA fragments together with a marker gene into the bacterial cells, and obtained deletion mutants through allelic exchange (Li et al., 2017), which is tedious and frequently troubled with low efficiency. Giving that *R. solanacearum* is a natural transformable bacterial species (Coupat et al., 2008; Coupat-Goutaland et al., 2011), we thought that this potency could be utilized to develop a simple and efficient method for gene deletion in *R. solanacearum.* To evaluate the potential of exploiting natural transformation for gene deletion in *R. solanacearum,* we firstly determined the transformation frequency of natural transformation by comparison with other two commonly used transformational approaches, i.e., triparental mating and electroporation (Figure 2A). To this end, suicide plasmid pK18mobsacB (ATCCR87097TM; Schäfer et al., 1994; Li et al., 2017), which is about 5.6 kb in size, and contains the gentamicin (Gm) antibiotic resistance gene, was purified for transformation of *R. solanacearum* strain EP1, which is tolerant to rifampin (Rif). After transformation, same amount of bacterial cells were spread on the CTG plates supplemented with Rif or Rif plus Gm, respectively, for counting total *R. solanacearum* EP1 colony forming units (CFU), and the number of colonies resistant to Gm (transformants). The potential transformants were further confirmed by PCR validating the presence of the Gm resistance gene. The results showed that the frequency of natural transformation was significantly higher than that of electroporation or triparental mating (Figure 2A). We then tested the influence of medium on the efficiency of DNA fragments natural transformation, and found that strain EP1 cultured in MM supplemented with 10% glycerol (MMG) displayed the highest transformation competence than that grown in LB or CTG (Figure 2B). The latter two media were normally used by us in preparation of competent cells of *Escherichia coli* or *R. solanacearum* for triparental mating and electroporation. In the DNA fragment concentration range (50 ng – 2 μg, in a final volume of 100 μl cells) used in this study, we found that the efficiency of natural transformation was enhanced along with the increased DNA level (Figure 2C). The results showed that the DNA concentration of positive transformants was low, about 500 ng.

### *Ralstonia solanacearum* gene knockout *via* natural transformation of a PCR product

Considering that construction of recombinant plasmid for transformation requires extra steps including DNA cloning in vector plasmid, transformation in *E. coli*, and plasmid purification, we set to test using PCR-generated DNA fragments for gene deletion in *R. solanacearum* through natural transformation. A previous study indicated that linearization of DNA could ensure higher transformation efficiency than circular plasmid (Bertolla et al., 1997), which was verified in our experiment (Figure 2A). In other naturally transformable bacterial species, such as *E. coli* and *Aliarcobacter butzleri*, natural transformation of PCR-generated fragments containing an antibiotic resistance marker was shown to be convenient for both chromosomal insertion and gene deletion applications (Komiyama and Maeda, 2020; Bonifácio et al., 2021). To verify this approach in *R. solanacearum* EP1, we selected two target genes, *phcA* and *epsB*, which are located on the circular chromosome and the mega plasmid, respectively. The *phcA* gene encodes a LysR family transcriptional regulator, controls the expression of many virulence genes, including the genes associated with EPS production (Schell, 2000). The *epsB* gene is essential for biosynthesis of extracellular polysaccharide, and *epsB*-deleted mutant failed to produce EPS I (Mori et al., 2018; Hayashi et al., 2019). To generate the fusion DNA fragments for transformation, we PCR-amplified the 5′- and 3′-flanking regions of the target genes as illustrated in Figures 1A,B, which were assembled together with the Gm resistance marker gene by fusion PCR, respectively (Figure 1C). The PCR fragments were then purified and introduced into strain EP1 through natural transformation, and integrated into the genome of strain EP1 through allelic exchange. The potential mutants were selected on CTG plates containing Rif and Gm, verified by PCR amplification (Figure 1D), and DNA sequencing (Supplementary Figure S1). Using this method, we found that the frequency of *phcA* and *epsB* deletion were at about 10 (1E-08) to 12 (1E-08), respectively, which were significantly higher than that achieved through plasmid-based triparental mating (Supplementary Figure S2).

**Figure 3:**
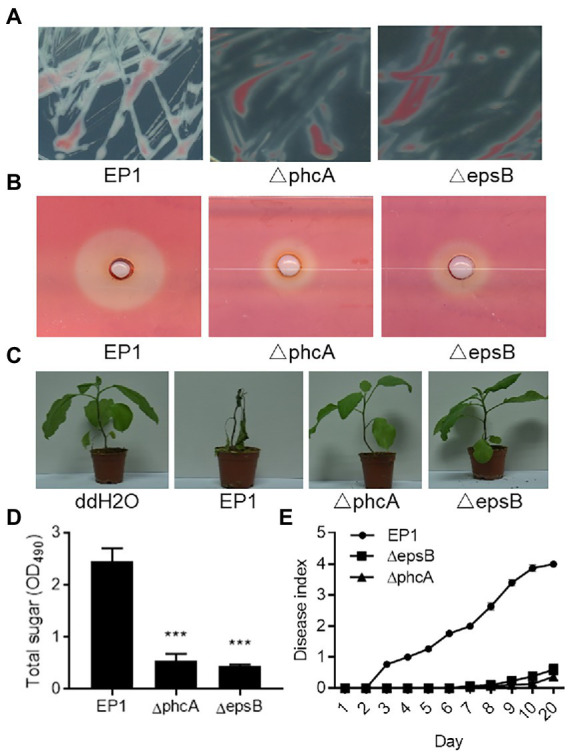
Phenotype validation of *Ralstonia solanacearum* strains EP1 and *phcA* and *epsB* mutants. **(A)** Bacterial morphology of *R. solanacearum* strains EP1 and *phcA* and *epsB* mutants on CTG medium. **(B)** The cellulase activity plate assay. The experiment was repeated three times with similar results. **(C)** Virulence assay on eggplants. The experiment was repeated three times with 10 plants per treatment each time. **(D)** Quantification of bacterial extracellular polysaccharides by measuring total sugar content. The experiment was repeated three times and data shown are means with standard deviation (SD). Statistical significance: ^***^, *p*-value < 0.001 (unpaired *t*-test). **(E)** Disease index of eggplants inoculated with *R. solanacearum* strain EP1 wild type and mutants. Plant disease symptoms were rated daily on a 0 to 5 disease index scale, where 0  = no symptom, 1–5 indicates 20%, 40%, 60%, 80%, and 100% plant leaves showing wilt symptoms. The experiment was repeated four times with 10 plants per treatment each time.

Deletions of the *phcA* and *epsB* genes were also validated by phenotype analysis. Similar to the previous reports (Genin and Denny, 2012; Perrier et al., 2018; Hayashi et al., 2019), deletion of *phcA* and *epsB* caused much decreased EPS production (Figures 3A,D), reduced cellulase activity (Figure 3B), and attenuated pathogenicity (Figures 3C,E).

### Generation of marker-free deletion mutants in *Ralstonia solanacearum* using the FLP/*FRT* system

**Figure 4:**
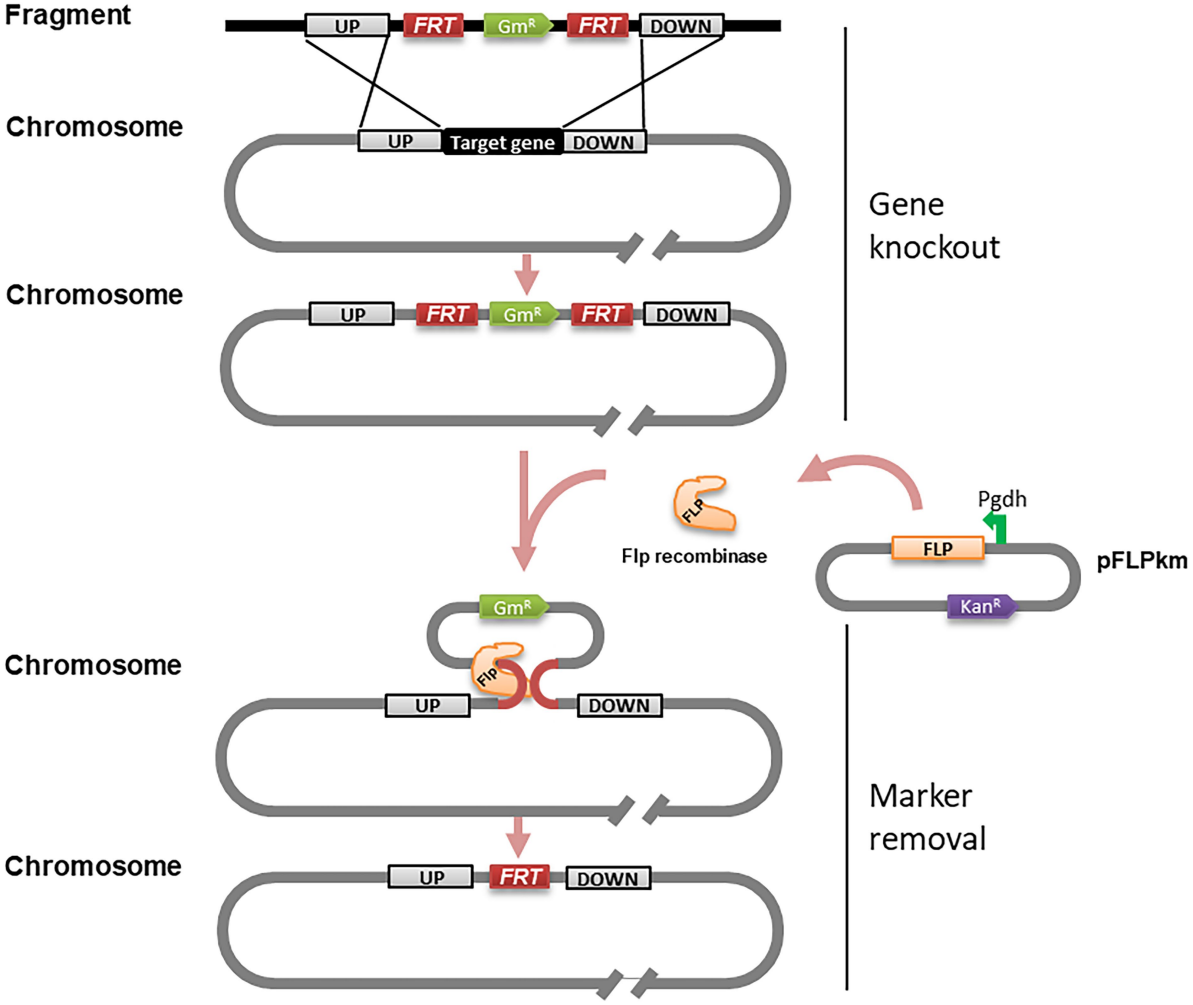
Schematic presentation of gene knockout and marker removal of the PCR fragment-based marker-frees gene deletion method for *Ralstonia solanacearum.* PCR fragments used in this experiment were identical to those illustrated in Figure 1A except that two 34 bp *FRT* sequences were incorporated to flank the maker gene Gm^R^. Symbol: *FRT*, a site sequence specifically recognized by FLP recombinase; Gm^R^, gentamicin resistance gene; UP, left flanking region; DOWN, right flanking region; pFLPkan, a plasmid containing FLP recombinase gene; Pgdh, a strong constitutive promoter; Kan^R^, kanamycin resistance gene.

Giving that double or even multiple gene deletion becomes common in functional analysis (Chen et al., 2020), a marker-free gene knockout method would be useful to overcome the limited availability of antibiotic resistance markers and minimize potential influence of heterologous genes. For this purpose, we adapted the FLP/*FRT* recombination system to remove the antibiotic resistance marker. FLP/*FRT* is a site-directed recombination system in which flippase (Flp) binds to both 13-bp 5′-GAAGTTCCTATTC-3′ arms flanking a 8-bp spacer in reverse orientation and cleavage occurs at the borders of the 8-bp spacer (Zhu and Sadowski, 1995). According to this principle, we generated the PCR fragments as above (Figure 1C), but added two *FRT* site sequences flanking the Gm resistant maker gene (Figure 4). The resulting PCR products were introduced into strain EP1 through natural transformation. The potential *phcA* and *epsB* knockout mutants were selected on CTG plates containing Rif and Gm, and confirmed by PCR analysis (Figure 1D). We selected validated knockout mutants of *phcA* and *epsB,* designated as △*phcA-Gm^R^* and △*epsB-Gm^R^* (Supplementary Figures S3A,B), respectively, for further marker removal analysis.

To excise the Gm resistance marker gene, we prepared an expression construct pFLPkm by cloning the *flp* gene under the control of the promoter P*gdh* in the plasmid vector pBBR1-MCS2 (Supplementary Figure S4; Figure 4). The purified pFLPkm was transformed into the knockout mutants △*phcA-Gm^R^* and △*epsB-Gm^R^,* respectively, by electroporation. The transformants containing pFLPkm were selected on CTG plates containing Km, and from which we randomly picked up 24 colonies derived from the △*phcA-Gm^R^* and △*epsB-Gm^R^* mutant lines (Supplementary Figures S3C,D) to validate the excision of the Gm marker gene by PCR, analysis (Figure 1D) and losing Gm resistance check on CTG plates containing Gm (Supplementary Figures S3E,F). The results showed that all the tested mutant colonies harboring pFLPkm lost the resistance and Gm-resistance marker, and all the control mutants without pFLPkm still contained the Gm-resistance marker. The plasmid pFLPkm in the mutants was then easily cured by passaging the bacterial cells in the culture medium without antibiotics.

### Gene deletion efficiency in *Ralstonia solanacearum* strains from different host plants

**Figure 5:**
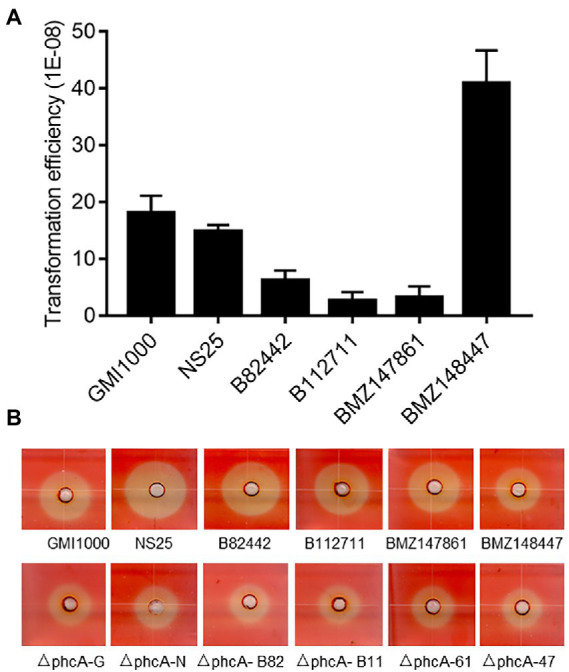
Marker-free gene deletion analysis of different *Ralstonia solanacearum* strains. **(A)** Natural transformation efficiency of *R. solanacearum* strains from different host plants: GMI1000 (tomato), NS25 (*Casuarina equisetifolia*), B82442 (potato), B112711 (tomato), BMZ147861 (tobacco), and BMZ148447 (zucchini). Data shown are means (± SD) of three biological replicates. **(B)** Cellulase activity plate assay of wild type strains and their *phcA* deletion mutants. The experiment was repeated three times with similar results.

Genome heterogeneity is common for *R. solanacearum* strains from different host plants, which may affect their natural transformation competence. To test the applicability of the gene deletion in the isolates from varied plant hosts, we utilized this PCR-fragment-based natural transformation method to delete the *phcA* gene in *R. solanacearum* strains GMI1000 (tomato), NS25 (*Casuarina equisetifolia*), B82442 (potato), B112711 (tomato), strain BMZ147861 (tobacco), and BMZ148447 (zucchini). The mutants showing typical phenotype changes were obtained from all tested *R. solanacearum* strains and confirmed by PCR analysis (Supplementary Figure S5) with varied efficiencies (Figure 5A). And deletion of *phcA* resulted in reduced cellulase activity (Figure 5B). The results indicate that the method developed in this study can be used in *R. solanacearum* strains from different host plants to generate marker-free deletions.

## Discussion

*Ralstonia solanacearum* species complex (RSSC) is a family of important plant pathogens with numerous hypothetical genes uncharacterized (Ailloud et al., 2015; Li et al., 2016). In addition, the renowned genome diversity of RSSC further increases the workload of post-genome studies (Geng et al., 2022). An efficient method for gene knockout or deletion could significantly facilitate functional analysis of the putative genes associated with the bacterial physiology and virulence. In this study, we developed a PCR fragment-based marker-free gene deletion method for RSSC by exploiting its natural transformation potency. We firstly compared the plasmid-based transformation frequencies of three methods, i.e., triparental mating, electroporation and natural transformation, in *R. solanacearum*. Triparental mating and electroporation are the two conventionally used methods for genetic transformation and gene deletion in RSSC (Li et al., 2017; Corral et al., 2020; Takemura et al., 2021). Our results showed that natural transformation offered a significantly higher level of transformation frequency than the other two methods using plasmid DNA (Figure 2A). Similarly, natural transformation could uptake and integrate the homologous PCR fragments containing an antibiotic-resistance gene into the bacterial genome and generate knockout mutants with a high frequency (Figure 1), which is agreeable with the previous findings that *R. solanacearum* is a naturally transformable bacterial species and capable of genetic recombination (Bertolla et al., 1997; Coupat et al., 2008). By combination use of the FLP/*FRT* system (Volkert and Broach, 1986), we showed that the integrated antibiotic resistance gene could be effectively eliminated to generate the marker-free deletion mutants. This feature could avoid potential influence of foreign genes/proteins and facilitate multiple gene deletion by recycle use of antibiotic resistance makers.

This study was built up on the several key findings from a previous study on the natural transformation competence of *R. solanacearum* (Bertolla et al., 1997): (1) the natural transformation competence was developed during exponential growth; (2) linear DNAs with a minimum of 50 bp homologous DNA could be effectively integrated into the bacterial genome of by recombination; and (3) minimum medium permits higher transformation frequency than rich medium. These findings have been exploited to integrate a reporter gene in the *R. solanacearum* genome to probe promoter strength and gene expression patterns (Monteiro et al., 2012). In that study, the reporter gene flanked by homologous DNA fragments was cloned in a suicide vector, transferred into *E. coli*, purified, linearized, purified again, and then introduced into *R. solanacearum* cells through natural transformation. To avoid these tedious procedures, we generated homologous DNA fragments containing an antibiotic resistance marker gene through fusion PCR, which were then used to transform *R. solanacearum.* The whole procedure involved only three steps, i.e., PCR, product purification, and transformation. In addition to gene deletion, as proven in this study, it is foreseeable that the simple and efficient method established in this study could also be used or modified for multiple gene deletion, promoter probing, gene integration, and gene overexpression etc. In this regard, it is interesting to note that *R. solanacearum* was able to exchange large DNA fragments ranging from 30 to 90 kb by DNA replacement (Coupat et al., 2008).

To validate this PCR fragment-based gene deletion method, we investigated the roles of *epsB* and *phcA* in the pathogenesis of *R. solanacearum* EP1, which is a virulent strain isolated from eggplant in South China (Li et al., 2016). Among them, *epsB* encodes a key enzyme essential for biosynthesis of EPS I, which is a key virulence factor of *R. solanacearum* (Hayashi et al., 2019), and *phcA* encodes a global regulator which plays a central role in the Phc quorum sensing system (Schell, 2000). In addition to regulate EPS I production, PhcA also regulates other virulence traits including plant cell wall degradation enzymes and the bacterial motility (Schell, 2000; Poussier et al., 2003). While the biological role of EpsB is highly conserved as a key enzyme for EPS I production, functional alternations in PhcA could be caused by certain growth conditions or phage infections (Poussier et al., 2003; Addy et al., 2012). We showed that deletion of *epsB* and *phcA* in *R. solanacearum* EP1 resulted in significantly decreased EPS production (Figures 3A,D), substantially reduced cellulase activity (Figure 3B), and much attenuated bacterial virulence against eggplants (Figures 3C,E). These results suggest that the virulence regulatory mechanisms of the Phc quorum sensing system are highly conserved in *R. solanacearum* EP1. Our recent study unveiled a new LuxIR type quorum sensing system, designated as RasIR, which plays a key role in regulation of virulence factor production in this pathogenic strain. It is highly intriguing how Phc and RasIR quorum sensing systems could be coordinated in modulation of the pathogenesis in this bacterial pathogen.

Although the natural transformation competence of RSSC strains has long been recognized and demonstrated (Boucher et al., 1985; Bertolla et al., 1997), it is far from clear whether all the *R. solanacearum* strains have similar transformation potencies. A previous report used 55 strains isolated from different hosts and geographical regions and found that 80% of strains distributed in all the phylotypes were naturally transformable by plasmids and/or genomic DNA (Coupat et al., 2008). To test the usability, we utilized this PCR-fragment-based natural transformation method established in this study to delete the *phcA* gene in *R. solanacearum* EP1 and other 6 strains, which are isolated from eggplant, tomato, potato, tobacco, zucchini and the forest tree *Casuarina equisetifolia,* respectively. The resultant marker-free deletion mutants showing typical phenotype changes were obtained from all tested *R. solanacearum* strains, but the transformation frequencies were varied with strain BMZ148447 isolated from zucchini showing the highest transformation frequency (Figure 5). It was speculated that variation in transformation frequencies may be related to the activity of the methyl mismatch repair system, or the transformation protocol used, which may be optimal for some strains but less ideal for other strains (Coupat et al., 2008). In this regard, it is interesting to note that the poorest transformation frequency achieved with this natural transformation method remained higher than that of triparental mating and comparable with that of electroporation (Figures 2A, 5). Taken together, the accumulated evidence suggests that natural transformation based genetic manipulation procedures shall have a wide applicability for functional analysis of the RSSC complex.

## Data availability statement

The original contributions presented in the study are included in the article/Supplementary material, further inquiries can be directed to the corresponding authors.

## Author contributions

JY, LZ, and LL conceived the study. JY did most of the experiments. NL, XW, XC, HW, QL, and XZ provided technical assistance. JY, LZ, and LL analyzed the data. LZ and LL supervised the study and wrote the manuscript with input from JY. All authors contributed to the article and approved the submitted version.

## Funding

This work was supported by the grants from Guangdong Forestry Science and Technology Innovation Project (2018KJCX009 and 2020KJCX009), the Key Realm R&D Program of Guangdong Province (2020B0202090001 and 2018B020205003), National Natural Science Foundation of China (31900076), Basic Research and Applied Basic Research Program of Guangdong Province (2020A1515110111 and 2022A1515010564), and Guangzhou Basic Research Program (202102020853).

## Conflict of interest

The authors declare that the research was conducted in the absence of any commercial or financial relationships that could be construed as a potential conflict of interest.

## Publisher’s note

All claims expressed in this article are solely those of the authors and do not necessarily represent those of their affiliated organizations, or those of the publisher, the editors and the reviewers. Any product that may be evaluated in this article, or claim that may be made by its manufacturer, is not guaranteed or endorsed by the publisher.
