# Supplementary material for: Markerless gene deletion in Ralstonia solanacearum based on its natural transformation competence
Source: Front Microbiol. 2022 Sep 13;13:977580. doi: 10.3389/fmicb.2022.977580 (PMC9512648; doi:10.3389/fmicb.2022.977580)
Supplement: Supplementary file 1 [file Data_Sheet_1.PDF]

Supplementary Materials

Title：Markerless gene deletion in *Ralstonia solanacearum* based on its natural transformation competence

Authors: Jinli Yan[1, 2], Nuoqiao Lin[1, 2], Xiaoqin Wang[1, 2], Xuemei Chen[1, 2], Huishan Wang[1, 2], Qiqi Lin[1, 2], Xiaofan Zhou[1], Lianhui Zhang[1, 2]*, Lisheng Liao[1]*

Content: Table S1 - S2

Figures S1 – S6

Table S1. Strains and plasmids used in this study

Strains/ Plasmids	Characteristics	Source or reference
**Strains**		
EP1	*R. solanacearum* EP1, wild type strain, Rif^R	(1)
GMI1000	*R. solanacearum* GMI1000, wild type strain, Rif^R	(2)
NS25	*R. solanacearum* NS25, wild type strain	This study
B82442	*R. solanacearum* B82442, wild type strain	This study
B112711	*R. solanacearum* B112711, wild type strain	This study
BMZ147861	*R. solanacearum* BMZ147861, wild type strain	This study
BMZ148447	*R. solanacearum* BMZ148447, wild type strain	This study
ΔepsB-Gm^R	The *epsB* Gm marker gene deletion mutant of strain EP1, Rif^R	This study
ΔphcA-Gm^R	The *phcA* Gm marker gene deletion mutant of strain EP1, Rif^R	This study
ΔepsB	The *epsB* gene deletion mutant of strain EP1, Rif^R	This study
ΔphcA	The *phcA* gene deletion mutant of strain EP1, Rif^R	This study
ΔphcB	The *phcB* gene deletion mutant of strain EP1, Rif^R	This study
E. coli DH5α	80dlacZΔM15Δ(lacZYA-argF)U169 recA1 endA1 hsdR 17 supE44 thi-1 gyrA relA1	Transgen
**Plasmids**		
pFLPkm	pBBR1MCS2 with *FLP*, between *EcoR*I and *Hind*III sites, Km^R	This study
pBBR1MCS2/5	Broad-host-range cloning vector , Km^R/Gm^R ,	This study
pRK2013	Tri-parental mating help plasmid, Km^R	This study
pK18mobsacB	Suicide and narrow-broad-host vector, Gm^R	This study

LITERATURE CITED

1. Li, P., D. Wang, J. Yan, J. Zhou, Y. Deng, Z. Jiang, B. Cao, Z. He, and L. Zhang. (2016). Genomic Analysis of Phylotype I Strain EP1 Reveals Substantial Divergence from Other Strains in the Ralstonia solanacearum Species Complex. Front Microbiol 7:1719.

2. Salanoubat, M., S. Genin, F. Artiguenave, J. Gouzy, S. Mangenot, M. Arlat, A. Billault, P. Brottier, J. C. Camus, L. Cattolico, M. Chandler, N. Choisne, C. Claudel-Renard, S. Cunnac, N. Demange, C. Gaspin, M. Lavie, A. Moisan, C. Robert, W. Saurin, T. Schiex, P. Siguier, P. Thebault, M. Whalen, P. Wincker, M. Levy, J. Weissenbach, and C. A. Boucher. (2002). Genome sequence of the plant pathogen Ralstonia solanacearum. Nature 415:497-502.

Table S2. Primers used in this study.

Primers	Characteristics*	Source or usage
phcA-1	5'-TGAACCACGGCACCTACAA	Deletion of *phcA*
phcA-2	5'-TAGGAACTTCTGGCTCAGACAGAAGGTGGA	
phcA-3	5'-GTCTGAGCCAGAAGTTCCTATTCTCTAGAAAGTATAGGAACTTCGGACGCACACCGTGGAAA	
phcA-4	5'-GAGGTTGTAGGTGAAGTTCCTATACTTTCTAGAGAATAGGAACTTCGGCGGCGTTGTGACAATTT	
phcA-5	5'-GGAACTTCACCTACAACCTCAACGCC	
phcA-6	5'-GCAGACCTCAAGAACATCG	
epsB-1	5'-CCATTCTTGTCGTGTGTATCG	Deletion of *epsB*
epsB-2	5'-TAGGAACTTCGCTGTTGGGATTGTCTT	
epsB-3	5'-TCCCAACAGCGAAGTTCCTATTCTCTAGAAAGTATAGGAACTTCGGACGCACACCGTGGAAA	
epsB-4	5'-GCGATAGGTATTGGGGAAGTTCCTATACTTTCTAGAGAATAGGAACTTCGGCGGCGTTGTGACAATTT	
epsB-5	5'-ACTTCCCCAATACCTATCGCTATGG	
epsB-6	5'-CCTTCATTGAGCAGGTTG	
Gen-F	5'-GGACGCACACCGTGGAAA	*Gen* gene detection primer
Gen-R	5'-GGCGGCGTTGTGACAATTT	
phcA-check-F	5'-AACAAGGTGCTGGAAATGG	*PhcA* gene knockout detection primer
phcA-check-R	5'-AACAGCCGCCTCATCAAA	
epsB-check-F	5'-GATTGAACGCACGGAATAC	*EpsB* gene knockout detection primer
epsB-check-R	5'-CCGAATACAACCAGGACTTT	
MCS-F	5'-GGCTTTACACTTTATGCTTCCG	*FLP* gene detection primer
MCS-R	5'-TTCCATTCGCCATTCAGG	
pK18-F	5'-CCCAGGCTTTACACTTTATGC	pK18mobsacB plasmid detection primer
pK18-R	5'- AACTGTTGGGAAGGGCGAT	

* Red highlights indicate FRT sequence, FLP recombinase recognition site. Underline indicate overlapping nucleotides.

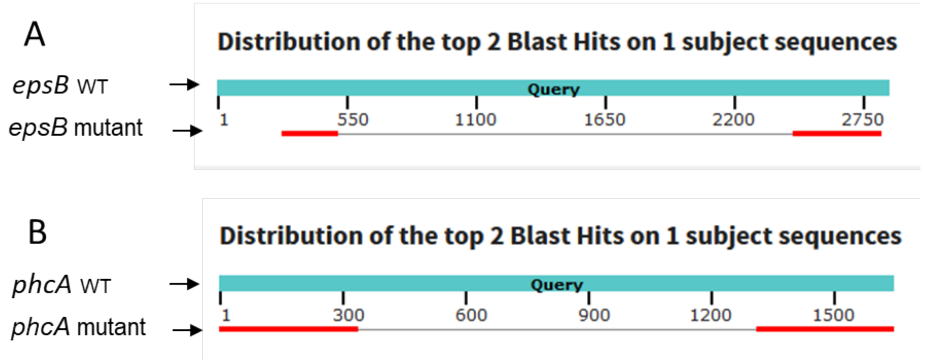

Figure S1. Sanger sequencing check and BLAST (https://blast.ncbi.nlm.nih.gov/Blast.cgi) sequence of *epsB* (A) and *phcA* (B) mutants with wild type.

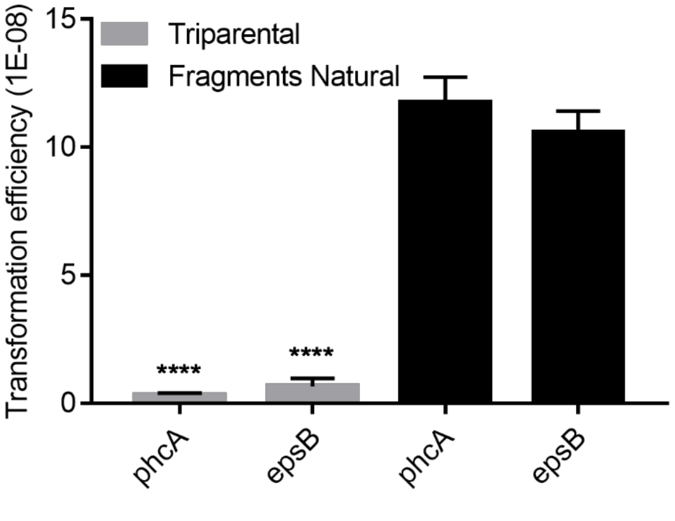

Figure S2. Triparental mating and fragments natural transformation efficiency assay of *phcA* and *epsB* deletion in *R. solanacearum* strain EP1. The amount of plasmid and DNA used in the experiment was approximately 2 μg. Transformation frequency was calculated as the number of transformants observed per *R. solanacearum* cell applied. Data shown are means ± standard deviations (SD) of three independent experiments with triplicates. Statistical significance: ****，P value < 0.0001 (unpaired t-test)

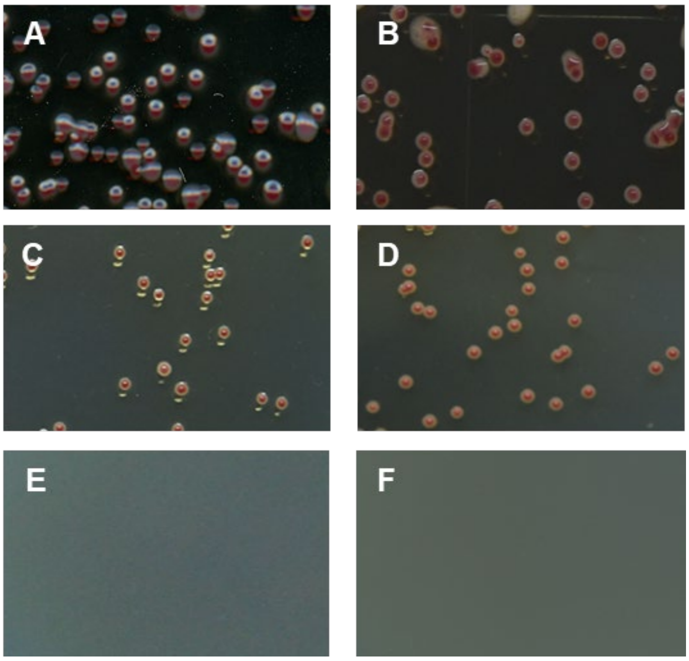

Figure S3. Generation of marker-free deletion mutants in *R. solanacearum*. △phcA-GmR (A) and △epsB-GmR (B) on CTG agar containing gentamicin were selected for marker removal analysis. pFLPkm were introduced into △phcA-GmR and △epsB-GmR by electroporation, respectively. The transformants containing pFLPkm were selected on CTG plates containing kanamycin for △phcA (C) and △epsB (D) Gm-resistance marker removal. To validate losing of the Gm resistance, △phcA (E) and △epsB (F) Gm-maker free mutants were check on CTG agar containing gentamicin, and both of mutants were failed to grow on the plates.

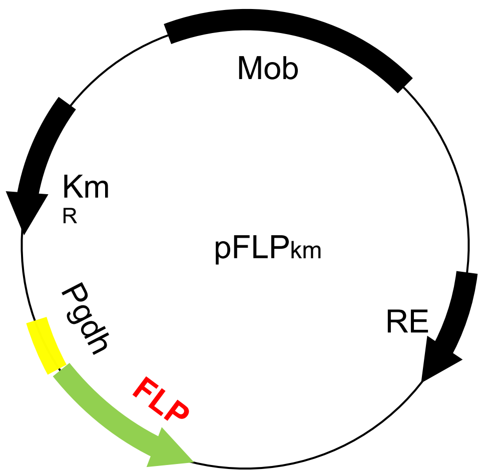

Figure S4. Genetic map of the plasmid $pFLP_{km}$. Pgdh is a strong promoter for driving *flp* gene.

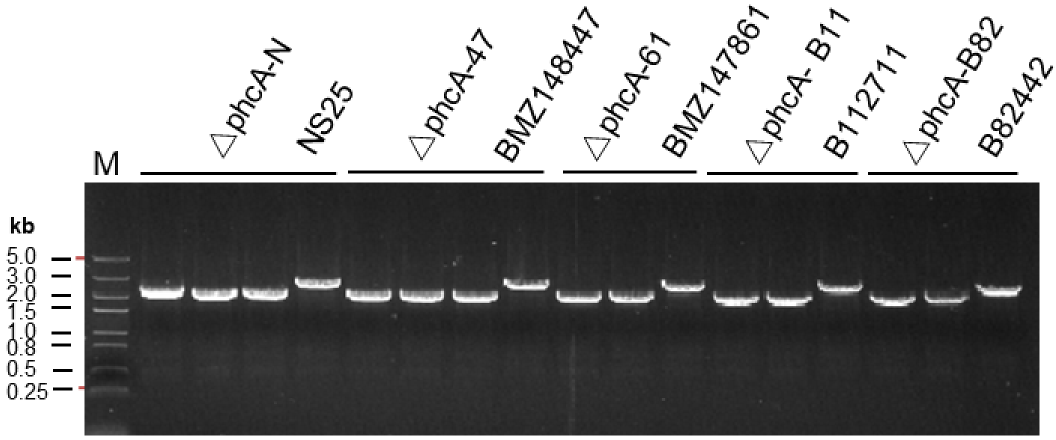

Figure S5. PCR analysis for validation of *phcA* deletion in 5 *R. solanacearum* strains from different host plants: NS25 (*Casuarina equisetifolia*), B82442 (potato), B112711 (tomato), BMZ147861 (tobacco), and BMZ148447 (zucchini).

phcA

phcA-check-F

CTACTCGAACAAGGTGCTGGAAATGGCCCGCGTGGTGGCCAAGTAAGCCTGCGCTTTTGCG

CAAAGGGAAAACGCGCCTGCGGGCGCGTTTTTTCATGCCGCGCAAGGTTGGACCATCGGG

GCGCCGTGCAAGCAAAGTGATGCAGCGGTTTCTTCGCGCGGGCGGTGGGGCGGATCGATG

TGATTCGGCCTGCCGCCCTTTGTTATGCACTGAAACGAAAACGTTTGCGTCTAAGCAAATTT

CGCAGACGGGCTGCTGGAATTCGCTTTGAAAATCGTCCGGCAATCCGTACACTGGACAATT

ACTACATTTGTGACGCAGTCGCCGTATCCTGCTGCGTCTGGGCCGTTGCCTCGGCAACGTCT

GCCTTTCCACCTTCTGTCTGAGCCAAAGCGTCCC|||||||||||||||||||||||||||||||

|||||||||||||||||||||||||||||||||||||||||||||||||||||||||||||||||||||

||||||||||||||||||||||||||TGCCAGCACCGCGCTCCTCCAACGAAAACGGCCGCAG

TGGATGCGGCCGTTTTTTTTGATGAGGCGGCTGTTGCGTCAGCGCTTGGGCCGGTGCGGGC

phcA-check-R

epsB

epsB-check-F

GATCGAGTCCGGTTGATTGAACGCACGGAATACGACCCTG|||||||||||||||||||||||

|||||||||||||||||||||||||||||||||||||||||||||||||||||||||||||||||||||

||||||||||||||||||||||||||||||||||CGGCTGCAGCGCGGCGAACCGATCGCCGCC

AGGGATTGCGAATCAGGAGGGGAATGGCGGTTGACAGCCGCCGGTTGAAGTCGCCGCCG

CAGCGCTGCCCACATCGGGCAGCGCTGCTGGTGTGTGAGTTCAGTCACGCATCCGTACCG

CAGAACGATGGGCCGGAGGCAAGGAGAGTCGCGCCGCGCGCGGGCCGATCCGGGCCGCG

GCCGGCGCAAGCGCGGAGTCGGTGGATCACACTATGAAAAAAGTCCTGGTTGTATTCGGT

epsB-check-R

Figure S6. The relative location of the primers phcA-check-F / phcA-check-R and epsB-check-F / epsR-check-R. Vertical lines indicate target gene sequences (no proportional), and PCR primers underlined.
